# Recurrent pseudogout after therapy with immune checkpoint inhibitors: a case report with immunoprofiling of synovial fluid at each flare

**DOI:** 10.1186/s40425-019-0597-x

**Published:** 2019-05-14

**Authors:** Sang T. Kim, Mohamad Bittar, Hyun J. Kim, Sattva S. Neelapu, Amado J. Zurita, Roza Nurieva, Maria E. Suarez-Almazor

**Affiliations:** 1 Departments of General Internal Medicine, The University of Texas MD Anderson Cancer Center, Houston, TX 77030 USA; 2 Department of Medicine, Section of Immunology, Allergy & Rheumatology, Baylor College of Medicine, Houston, TX 77030 USA; 3 The University of Cincinnati College of Medicine, Cincinnati, OH 45267 USA; 4 Department of lymphoma/myeloma, The University of Texas MD Anderson Cancer Center, Houston, TX 77030 USA; 5 Genitourinary Medical Oncology, The University of Texas MD Anderson Cancer Center, Houston, TX 77030 USA; 6 Department of Immunology, The University of Texas MD Anderson Cancer Center, Houston, TX 77030 USA

**Keywords:** Immunotherapy, Immune-related adverse events, Pseudogout, Th17 cells

## Abstract

**Background:**

Despite ground-breaking clinical success in the treatment of different cancers, immune checkpoint inhibitors can cause profound inflammatory and immune-related adverse events. Autoimmune inflammatory arthritis following immune checkpoint inhibitor treatment has been reported; however, to date, no cases of crystal arthritis following immune checkpoint inhibitors have been identified.

**Case presentation:**

We report the first case of recurrent pseudogout, an inflammatory crystal arthritis, in a patient treated with nivolumab, a PD-1 inhibitor, for renal cell carcinoma. The patient had recurrent pseudogout flares about week to 10 days after each nivolumab infusion. After treatment with prophylactic colchicine, the patient well tolerated additional nivolumab infusions without adverse events. In parallel, we characterized immune cells of synovial fluid at each flare. Immunoprofiling of synovial fluid showed that the proportion of inflammatory IL-17-producing CD4^+^ T cells and amount of IL-17 were notably increased in synovial fluid with every recurrent flair, and correlated with the increase in number of synovial neutrophils, suggesting a potential role of T helper 17 (Th17) cells in neutrophil-driven inflammation during pseudogout arthritis.

**Conclusions:**

This case suggests a potential influence of Th17 cells on the neutrophil recruitment and neutrophil-driven inflammatory events leading to pseudogout induced by immune checkpoint inhibitor therapy.

## Background

By targeting T cell inhibitory molecules and reinvigorating exhausted T cells, immune checkpoint inhibitors (ICIs) have opened the new chapter in cancer treatment [1]. Types of ICIs currently approved include cytotoxic T-lymphocyte-associated protein 4 (CTLA-4), programmed cell death-1 (PD-1), and programmed cell death ligand-1 (PD-L1) inhibitors [2]. Despite their clinical benefits, ICIs have distinct toxicity, causing a myriad of immune-related adverse events (irAE). Inflammatory arthritis following ICI therapy has been reported and is thought to be primarily autoimmune, although its etiology remains unclear [3–7], to date, no cases of crystal arthritis following ICI therapy have been reported.

Crystal arthritis are autoinflammatory disorders, and inflammasome and innate immune system play key roles in their pathogenesis [8]. Gout and pseudogout are the two most common types of crystalline arthritis. We report the first case of crystal-proven pseudogout after treatment with nivolumab, a PD-1 inhibitor. The patient had recurrent pseudogout flares after each nivolumab infusion. After initiating treatment with prophylactic colchicine, the patient well tolerated several additional nivolumab infusions. In parallel, we performed immunoprofiling of the synovial fluid obtained at each flare.

## Case presentation

A 63 year old male with renal cell carcinoma underwent nephrectomy in 2015. He was under active surveillance until 2017, when he presented with metastatic disease in lymph nodes and lungs. The patient received nivolumab combined with ipilimumab, a CTLA-4 inhibitor, for 3 months followed by nivolumab monotherapy at a dose of 250 mg every 2 weeks. After 6 months of treatment, he developed immune-related type I diabetes, starting insulin, and continued immunotherapy. After receiving 14 infusions of nivolumab, 253 days after the first infusion, he developed acute left knee pain and swelling and was referred to the rheumatology clinic. Patient denied any prior similar episodes, but had a remote history of injury to his left knee, decades before, with ligament damage, for which he underwent arthroscopy.

Past medical history included hypertension, hypothyroidism and stage 2 chronic kidney disease, all diagnosed prior to immunotherapy. He had no history of recent trauma. One of his children had gout.

Physical exam revealed a large effusion in the left knee with erythema, tenderness, and limited range of motion. Arthrocentesis was performed and synovial fluid analysis showed 6715 white blood cells per μL, 72% of neutrophils, and numerous intracellular and extracellular calcium pyrophosphate dihydrate (CPPD) crystals (Table 1). Gram, acid-fast bacilli (AFB), and fungal stains, and cultures were negative. Anti-nuclear antibody, rheumatoid factor, and anti-cyclic citrullinated peptide (CCP) antibody were negative. Knee x-ray showed tricompartmental degenerative changes without fractures or bony metastases. Although very subtle, the x-ray showed chondrocalcinosis of fibrocartilage, hyaline cartilage and suprapatellar bursal synovium. Patient received an intra-articular injection of 40 mg triamcinolone, with an excellent response. Nivolumab was held, and restarted 3 weeks later as the patient had complete resolution of his left knee arthritis. A few days after the infusion, the patient had another flare of left knee pain and swelling. Knee arthrocentesis followed by intra-articular triamcinolone injection was performed with an excellent clinical response within 2 weeks. Synovial fluid again showed CPPD crystals. Two weeks later, after complete resolution, nivolumab was resumed, however, once again, a few days later, he developed left knee arthritis with CCPD crystals in the synovial fluid, and patient received intra-articular triamcinolone. Of note, his thyroid-stimulating hormone (TSH) levels were elevated during the 2nd and 3rd pseudogout flares. Tumor staging showed stable disease, and the patient decided to continue nivolumab therapy despite the recurrent pseudogout flares. He initiated colchicine 0.6 mg orally daily as prophylaxis. After resolution of his knee symptoms, he resumed nivolumab treatment. Patient has continued nivolumab treatment with colchicine prophylaxis and has received three additional nivolumab infusions at the same dose without pseudogout flares. He is now receiving nivolumab 480 mg monthly with good tolerance and only mild pain after the infusions. As of last follow-up he had minimal knee effusion with no pain, tenderness, or limitation in the range of motion. He was able to ambulate and perform activities of daily living. He has had one additional episode of left knee arthritis, but much milder than previous ones, and no arthrocentesis was performed. His magnetic resonance imaging (MRI) of the knee showed primarily partial meniscal tears and damage to his anterior cruciate ligaments.

**Table 1:** Characterization of synovial fluid

	1st flare	2nd flare	3rd flare
White blood cells (cells/μL)	6715	18,000	12,130
Lymphocytes (%)	17	4	2
Neutrophil (%)	72	75	92
Histiocytes (%)	11	21	5
Bacteria culture	negative	negative	negative
Fungal culture	negative	n/a	n/a
AFB culture	negative	n/a	n/a
Crystals	Intra and extracellular CPPD	Intra and extracellular CPPD	Intra and extracellular CPPD

*AFB* acid-fast bacilli, *n/a* not available, *CPPD* Calcium pyrophosphate dihydrate

## Methods

### Isolation of cells

Synovial fluid of the left knee was collected at each pseudogout flare using standard sterile procedures, before receiving any treatment. Synovial fluid samples were incubated with 10 IU collagenase III (Sigma, Cat No: H3506) at 37 °C degrees for 15 min. After incubation, the sample was centrifuged at 500G for 10 min and the synovial fluid was collected. The remaining cells were washed with phosphate-buffered saline (PBS) (Gibco™) and cryopreserved in the presence of 90% fetal bovine serum (Gibco®, Cat No: 16140071) and 10% dimethyl sulfonoxide (Sigma®, Cat No: D2650).

### Flow cytometry

Cryopreserved synovial fluid cells were thawed, washed with complete RPMI-1640 medium containing 10% fetal bovine serum, glutamine, penicillin, streptomycin, and amphotericin B (Gibco®) and stained with flow cytometry antibodies. We performed intracellular staining to evaluate effector cytokines of CD4^+^ T cells. Cells were stimulated for 4 h in the presence of 1x cell stimulation cocktail containing phorbol 12-myristate-13-acetate, ionomycin, and brefeldin A (Biolegend®, Cat No: 423303) followed by staining of surface markers, fixation (BD CytoFix/CytoPerm™, Cat No: 51-2090KZ), permeabilization (BD PERM/ Wash™ solution, Cat No: 51-2091KZ), and intracellular cytokine staining. Stained samples were acquired by BD LSR II FORTESSA™ X-20 and analyzed with FlowJo software® (TreeStar, CA). Flow cytometry antibodies used in this study are following; LIVE/DEAD Zombie Aqua™ (BioLegend®), anti-CD16 BUV395 (3G8, BD Horizon™), anti-CD19 PE (HIB19, BioLegend®), anti-CD3 PerCP/Cyanine 5.5 (SK7, BioLegend®), anti-HLA-DR Alexa Fluor® 488 (L243, BioLegend®), anti-CD123 PE (6H6, BioLegend®), anti-CD11c PE-Cy7 (Bu15, BioLegend®), anti-CD14 Alexa Fluor® 700 (MSE2, BioLegend®), anti-TCR gamma/delta Brilliant Violet 421™ (B1, BioLegend®), anti-CD45RA Brilliant Violet 785™ (HI100, BioLegend®), anti-CD56 FITC (HCD56, BioLegend®), anti-CD19 Brilliant Violet 785™ (HIB19, BioLegend®), anti-CCR7 PE-Cy7 (G043H7, BioLegend®), anti-CD4 BUV395 (SK3, BD Horizon™), anti-CD8 Alexa Fluor® 700 (HIT8a, BioLegend®), anti-CD25 FITC (BC96, BioLegend®), anti-CXCR5 APC (J25D4, BioLegend®), anti-CD127 Alexa Fluor® 700 (A019D5, BioLegend®), anti-IL-4 Brilliant Violet 421™ (MP4-25D2, BioLegend®), anti-IL-21 PE (3A3-N2.1, BD Horizon™), anti-IFNγ PE/Dazzle™ 594 (4S.B3, BioLegend®), anti-IL-17A PE-Cy7 (BL168, BioLegend®).

### Enumeration of synovial immune cells

**Fig. 1:**
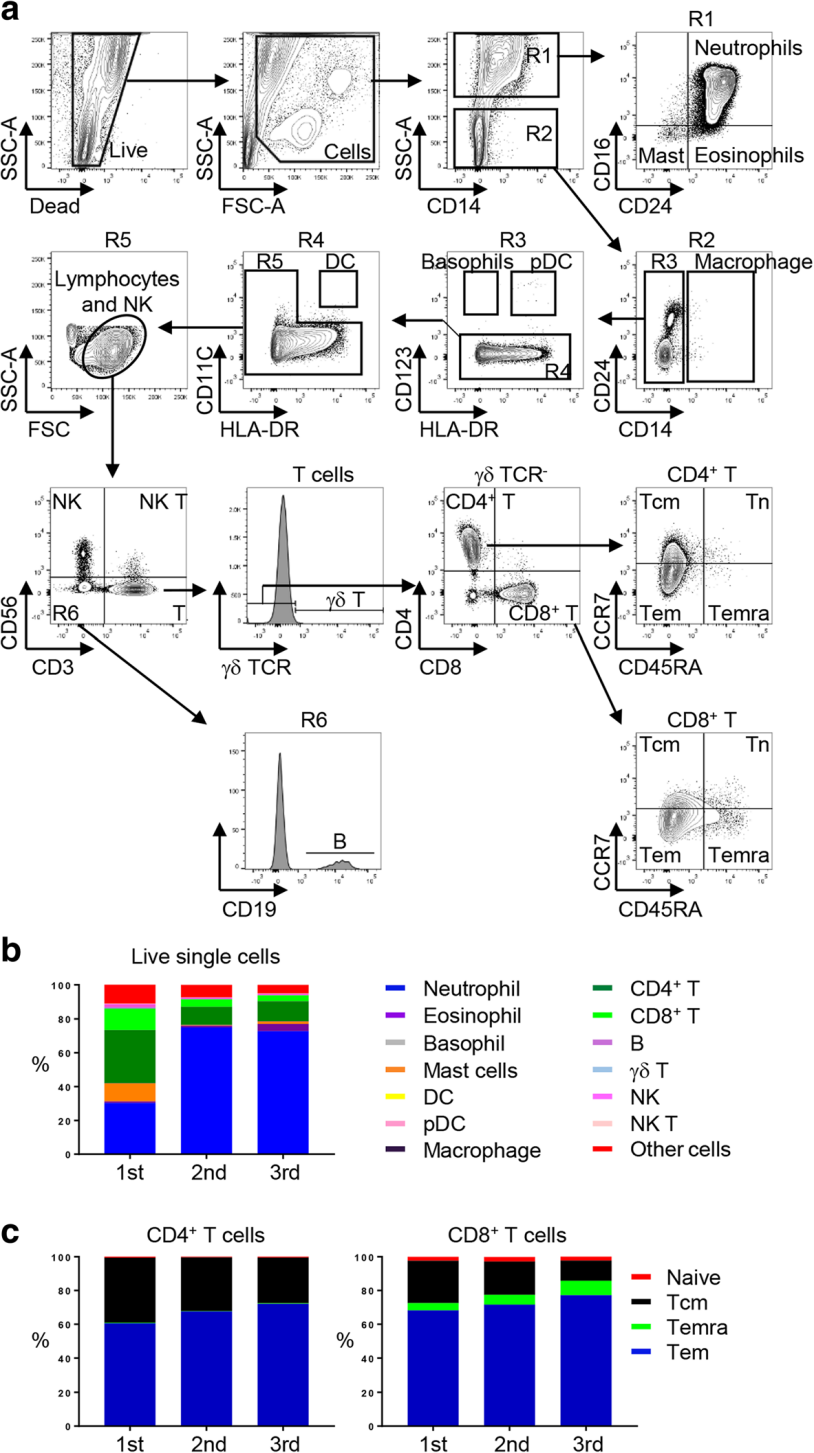
Flow cytometry analysis of synovial immune cells at each pseudogout flares. **a** Flow cytometry gating strategy of major immune cells. One of the most representative plots. FSC-A, forward scatter area; SSC-A, side scatter area; HLA-DR, human leukocyte antigen DR; Mast, Mast cells; Macro, Macrophages; pDC, plasmacytoid dendritic cells; NK, natural killer cells; NK T, natural killer T cells; γδ T, γδ T cells; CD4^+^ T, CD4^+^ T cells; CD8^+^ T, CD8^+^ T cells; B, B cells; Tcm, central memory T cells; Tn, naïve T cells; Tem, effector memory T cells; Temra, terminally differentiated T cells. **b** Percentage of major immune cell subsets within total live single cells. DC, dendritic cells; pDC, plasmacytoid dendritic cells; NK, natural killer cells; NK T, natural killer T cells. **c** Percentage of T cell subsets. Tcm, central memory; Tem, effector memory; Temra, terminally differentiated effector memory cells

To enumerate major immune cell subsets, we adapted and modified the gating strategy from the study by Yu et al. (Fig. 1a) [9]. We calculated proportions of CD4^+^ T cell subsets including CD45RA^+^ naïve, regulatory T cells (Tregs; CD25^hi^ CD127^lo^) [10], C-X-C chemokine receptor type 5 (CXCR5)^+^ follicular helper T cells, a distinct CD4^+^ T cell subset helping B cells produce immunoglobulins [11], and CD45RA^−^ CXCR5^−^ effector cells. We also enumerated CD4^+^ T cells producing effector cytokines including interferon gamma (IFNγ), interleukin (IL)-4, IL-17, and IL-21.

### Cytokine measurement

Cytokines in synovial fluid were measured by multiplex or classical ELISA techniques using commercially available kits (U-Plex Th17 Combo 2 and U-Plex Th1/Th2 Combo, both Meso Scale Discovery, LLC; IL-8 Human Uncoated ELISA kit, Invitrogen™), according to the manufacturer’s instructions.

## Results

First, we performed flow cytometry to characterize immune cell subsets of synovial fluid obtained at each time that the patient had a pseudogout flare (Fig. 1a). Consistent with findings in clinical settings, neutrophils were dominant in the synovial fluid especially in the second and third flares (Fig. 1b; 30.36, 75.00, and 72.80% within live single cells at each flare). Of note, CD4^+^ T cells were the most abundant lymphoid cells in all flares (Fig. 1b; 31.42, 10.79, and 11.63% within live single cells at each flare). The effector memory population was significantly increased within both CD4^+^ and CD8^+^ T cells, suggesting they may be involved in the development and progression of inflammation (Fig. 1c).

**Fig. 2:**
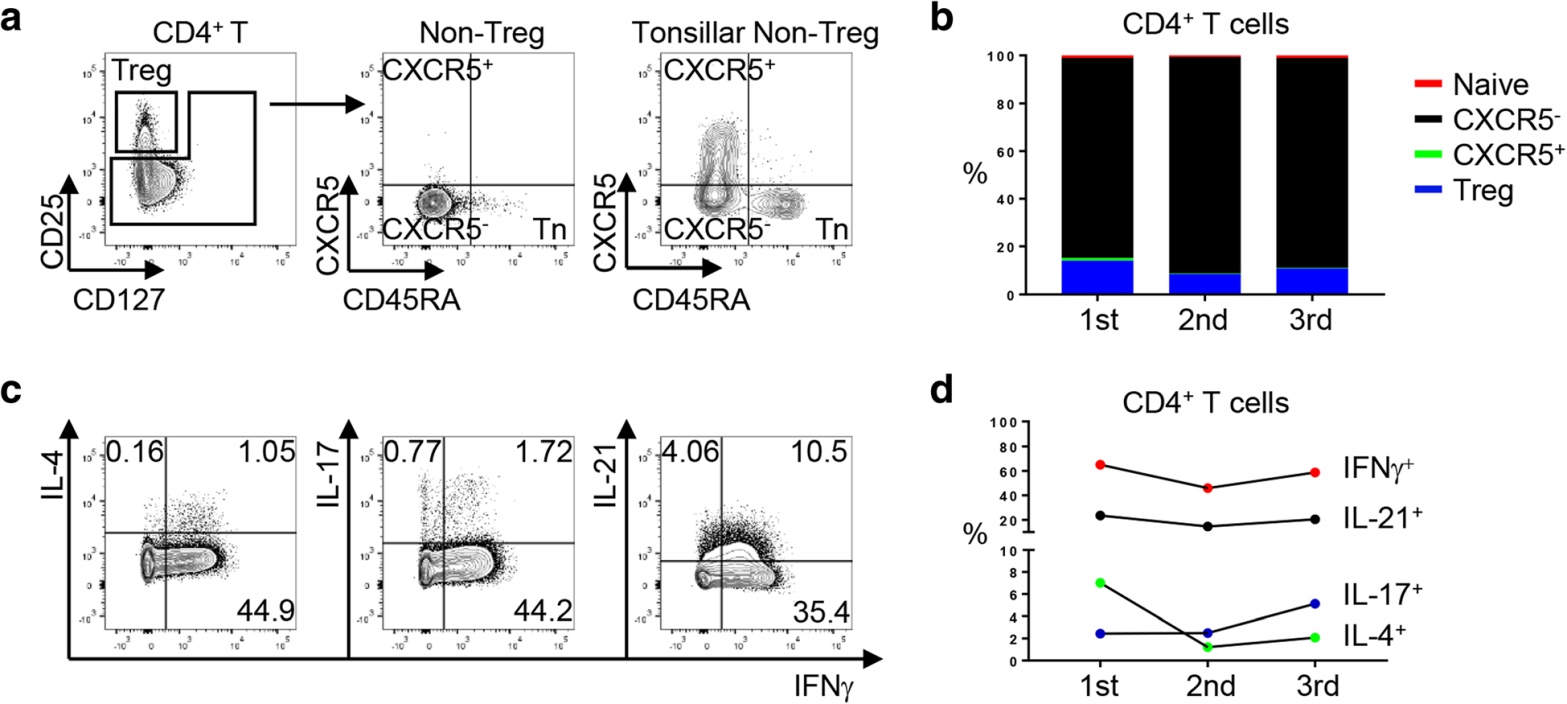
Flow cytometry analysis of synovial CD4^+^ T cells at each pseudogout flares. **a** Flow cytometry gating strategy of CD4^+^ T cells. One of the most representative plots. Gating of CD45RA and CXCR5 was made based on the expression of CD45RA and CXCR5 on anonymous tonsillar non-Tregs (right panel). Treg, regulatory T cells; Non-Treg, non-regulatory T cells; Tn, naïve T cells. **b** Percentage of CD4^+^ T cell subsets within CD4^+^ T cells. Treg, regulatory T cells. **c**-**d** Percentage of cytokine producing CD4^+^ T cells. One of the most representative plots (**c**) and quantitative analysis (**d**)

Given the central role of CD4^+^ T cells in immune responses, we focused on CD4^+^ T cells in synovial fluid (Fig. 2). Flow cytometry analysis of CD4^+^ T cell subsets in synovial samples revealed that the most abundant CD4^+^ T cell subset was CXCR5^−^ CD4^+^ T cells followed by Tregs (Fig. 2a-b). Naïve and CXCR5^+^ CD4^+^ T cells were detected at low frequency in synovial fluid. Intracellular staining of CD4^+^ T cells showed enhanced but sustained level of IFNγ producing CD4^+^ T helper 1 (Th1) cells during all three flares (Fig. 2c-d). Of note, IL-17 producing CD4^+^ T helper 17 (Th17) cells, the inflammatory CD4^+^ T cell subset known to recruit neutrophils via IL-17 were detected at first and second flairs and subsequently increased with the third flare (2.45, 2.49, and 5.12% within CD4^+^ T cells at each flare). Similarly, synovial fluid cytokines quantification assay also revealed substantial levels of IL-17 in first and second flares followed by a significant increase in the third one. In addition to IL-17, the synovial fluid contained significant levels of an inflammatory cytokine, IL-6, a key factor for Th17 differentiation and for neutrophil recruitment [12], and IL-8, a neutrophil chemoattractant (Table 2) [13].

**Table 2:** Cytokine concentration in synovial fluid

Cytokines (pg/ml)	1st flare	2nd flare	3rd flare
IL-1β	4.13	3.73	5.08
IL-6	3552.95	5042.89	6565.92
IL-8	523.96	98.58	439.32
TNFα	6.12	5.94	6.55
IFNγ	60.57	77.92	53.39
IL-17A	11.25	9.43	16.59
IL-21	2.50	4.65	6.82

*IFNγ* interferon γ, *TNFα* tumor necrosis factor α

## Discussion and conclusions

We report the first case of crystal-proven pseudogout after ICI treatment. After having received several infusions of nivolumab over 35 weeks with no articular adverse events, the patient developed recurrent pseudogout flares, about a week to 10 days after each infusion. Notably, with colchicine prophylaxis, he was able to tolerate subsequent infusions without acute recurrence, and only mild symptoms. The proportion of inflammatory IL-17-producing CD4^+^ T cells and amount of IL-17 were notably increased in synovial fluid with every recurrent flair, and correlated with the increase in number of synovial neutrophils, suggesting a potential role of Th17 cells in neutrophil recruitment and neutrophil-driven inflammatory pathways resulting in pseudogout arthritis induced by immune checkpoint inhibitor therapy.

We and others have reported cases of immune-related arthritis in patients receiving irAE [3–7]. This type of arthritis can involve both large and small joints, and is more commonly seen in patients receiving PD-1 inhibitors, rather than CTLA-4 inhibitors. Rheumatoid factor and anti-CCP antibody are often negative; however, it has been assumed that these cases may represent autoimmune events triggered by the up-regulation of the immune system induced by ICI [14]. To date no cases of crystal arthritis after ICI treatment have been reported. Most studies on the pathophysiology of crystal arthritis have studied gout, rather than pseudogout, but it is thought that the triggering inflammatory mechanisms are similar for both conditions. Crystal arthritis is primarily a neutrophil-driven autoinflammatory disorder with no apparent role for adaptive immunity [15], while ICI mechanism of action primarily reactivates T cells [1]. Thus, our case suggests a potential impact of inflammatory T cells on the initiation and progression of crystal arthritis in patients receiving ICI.

The cells mostly studied in crystal arthritis are neutrophils and macrophages. Macrophages sense and phagocytose crystals, activating NLRP inflammasome with subsequent secretion of pro-inflammatory cytokines, especially IL-1β and tumor necrosis factor (TNF) α [16–18]. In addition, CPPD crystals bind to Toll–like receptor (TLR) 2 and 4 that leads to NF-kB activation and release of cytokines, TNFα, IL-6, and IL-8. Secretion of IL-1β, TNFα, IL-6, and IL-8 by monocytes increases expression of adhesive molecules on endothelial cells that attract neutrophils to the site of crystal deposition [19]. Recently, Pang et al. showed that CPPD crystals induce formation of neutrophil extracellular traps (NETs) which are associated with both autophagy and IL-1β production [20]. Of note, neutrophil cytoplasts, detected only after the formation of NETs [21], induce Th17 differentiation in severe asthma [22]. It is plausible that macrophage and neutrophil derived cytokines such as IL-6 and IL-1β may have contributed in our patient to Th17 differentiation. In turn, through IL-17, Th17 cells enhance endothelial expression of neutrophilic chemokines C-X-C chemokine ligand (CXCL) 1 and CXCL2, and neutrophil influx to the sites of inflammation.

Interestingly, the patient successfully resumed nivolumab treatment while on colchicine prophylaxis. By inhibiting tubulin assembly and suppressing microtubule formation, colchicine inhibits cell proliferation, mainly in neutrophils in pseudogout [19]. Colchicine also inhibits formation of inflammasome and decreased IL-1β secretion by CPPD-stimulated macrophages [16]. In addition, colchicine may have had direct or indirect effects on T cell activation, differentiation and memory T cell responses.

Endocrine and metabolic diseases including diabetes, hypothyroidism, hyperparathyroidism, hypomagnesemia, hypophosphatemia, and hemochromatosis are known risk factors for pseudogout arthritis [23]. Interestingly, our patient had hypothyroidism prior to the immunotherapy, and immunotherapy-induced type I diabetes, which may have contributed to his pseudogout arthritis.

This is the first case reporting pseudogout arthritis after ICI. Crystal arthritis may occur more frequently in these patients, and may be underecognized by both oncologists and rheumatologists, as the attention has been primarily on de novo presenting as an irAE, with a possible autoimmune mechanism. We expect our report will lead to increased recognition of crystal arthritis in patients undergoing cancer immunotherapy, and be included in the differential diagnoses. Immunophenotypic analyses suggested contribution of a feed-forward loop between Th17 cells and neutrophils in the joint inflammation. Comprehensive analyses with additional cases and control samples will be needed to further describe this newly recognized ICI-induced adverse event.

## Abbreviations

AFB: Acid-fast bacilli
CCP: Cyclic citrullinated peptide
CPPD: Calcium pyrophosphate dihydrate
CTLA-4: Cytotoxic T-lymphocyte-associated protein 4
CXCL: C-X-C chemokine ligand
ICI: Immune checkpoint inhibitors
irAE: Immune-related adverse events
NETs: Neutrophil extracellular traps
PD-1: Programmed cell death-1
TLR: Toll-like receptor
TNF: Tumor necrosis factor
